# ETC-159, an Upstream Wnt inhibitor, Induces Tumour Necrosis via Modulation of Angiogenesis in Osteosarcoma

**DOI:** 10.3390/ijms24054759

**Published:** 2023-03-01

**Authors:** Kenon Chua, Arthur Yi Loong Sim, Eric Yew Meng Yeo, Muhammad Sufyan Bin Masroni, Wah Wah Naw, Sai Mun Leong, Kee Wah Lee, Huey Jin Lim, David M. Virshup, Victor Kwan Min Lee

**Affiliations:** 1 Programme in Cancer and Stem Cell Biology, Duke-NUS Medical School, Singapore 169857, Singapore; 2 Department of Orthopaedic Surgery, Singapore General Hospital, Singapore 169608, Singapore; 3 Programme in Musculoskeletal Sciences Academic Clinical Program, SingHealth/Duke-NUS, Singapore 169857, Singapore; 4 Department of Pathology, Yong Loo Lin School of Medicine, National University of Singapore, Level 3 NUH Main Building, 5 Lower Kent Ridge Road, Singapore 119074, Singapore; 5 Department of Anatomy, Yong Loo Lin School of Medicine, National University of Singapore, MD10, 4 Medical Drive, Singapore 117594, Singapore; 6 Program in Cancer and Stem Cell Biology, Duke-NUS Medical School, Singapore 169857, Singapore

**Keywords:** Wnt, osteosarcoma, ETC-159, angiogenesis

## Abstract

There is an increasing urgency in the search for new drugs to target high-grade cancers such as osteosarcomas (OS), as these have limited therapeutic options and poor prognostic outlook. Even though key molecular events leading to tumorigenesis are not well understood, it is widely agreed that OS tumours are Wnt-driven. ETC-159, a PORCN inhibitor that inhibits the extracellular secretion of Wnt, has recently progressed on to clinical trials. In vitro and in vivo murine and chick chorioallantoic membrane xenograft models were established to examine the effect of ETC-159 on OS. Consistent with our hypothesis, we noted that ETC-159 treatment not only resulted in markedly decreased β-catenin staining in xenografts, but also increased tumour necrosis and a significant reduction in vascularity—a hereby yet undescribed phenotype following ETC-159 treatment. Through further understanding the mechanism of this new window of vulnerability, therapies can be developed to potentiate and maximize the effectiveness of ETC-159, further increasing its clinical utility for the treatment of OS.

## 1. Introduction

High grade osteosarcomas (OS) are the most common primary bone malignancy. Typically affecting adolescents or children during their puberty growth spurt, the current standard therapy consists of complete surgical resection of the primary and metastatic tumours, in combination with neoadjuvant and adjuvant chemotherapy [1]. Despite advances in our understanding of the disease and various new promising therapeutic approaches [2], survival rates have stagnated at approximately 70 percent [1] over the last 30 years. Prognosis is especially poor for patients with metastatic, recurrent, or non-resectable disease, with 5-year overall survival rates at less than 30 percent [3,4,5]. This has led to the investigation of key signalling pathways implicated in the development of OS so as to develop new chemotherapy drugs.

Complicating the search for effective drugs for treatment is the fact that key molecular events that lead to tumorigenesis in OS are not well understood. Secondary changes in cell cycle regulation or DNA replication have been implicated [6]. These changes appear to lead to an abnormal accumulation of β-catenin within the nucleus [7], pointing to aberrant Wnt signalling as a possible pathway to be investigated for the development of therapeutic targeting. 

Wnts are a family of highly conserved signalling ligands responsible for developmental and tumorigenic processes [8]. This family of signalling ligands work in a synergistic manner [9] to elicit key downstream cellular responses involved in proliferation and differentiation. Wnts have also been shown to play a key role in the tumorigenesis of various cancers [10,11,12]. Specifically, Wnt signalling has been implicated in OS malignancy. Consistent with this, multiple previous studies have reported that OS is driven by high Wnt signalling [13,14,15,16,17]. Taken together, Wnt inhibition is therefore a promising therapeutic strategy for the treatment of OS.

The Wnt signalling pathway has also been shown to be necessary for the modulation of angiogenic response in oncogenesis [18,19,20]. Elevated levels of β-catenin have been noted to accumulate in tumorigenic tissues following aberrations in the Wnt signalling pathway. Stabilized β-catenin is then able to enter the nucleus and initiate the transcription of pro-angiogenic factors. These factors are important in regulating the migration, proliferation, and eventual differentiation of endothelial cells for the formation of blood vessels. Wnts therefore directly regulate vascular development [21], and previous literature has shown that high levels of VEGF, and therefore high angiogenesis in OS, is correlated with poor prognosis [22]. Angiogenesis is one of the hallmarks of cancer, and necessary for the formation of intratumoral blood vessels to supply nutrients and remove waste [23]. Inhibition of angiogenesis would lead to suppression of tumour growth [24].

In this study, we established fresh patient-derived OS cell lines to investigate if clinical samples of OS demonstrated high β-catenin and possess intact Wnt signalling in order to identify if this group of human cancers are potentially treatable by an upstream Wnt inhibitor. We then selected 2 well characterized OS cell lines (143B and SJSA-1) which had previously been published extensively [25,26,27] and treated them with upstream Wnt inhibitor ETC-1922159 (hereafter ETC-159) in a murine xenograft model to examine if Wnt inhibition could offer therapeutic opportunities of clinical benefit. The authors aim to characterize the response of ETC-159 in OS, and to go further by showing that the reduction in vasculature from inhibition of Wnt signalling leads to the clinically beneficial outcome of increased tumour necrosis. 

## 2. Results

### 2.1. Clinical Osteosarcoma Samples Demonstrate High WLS and β-Catenin Expression

**Figure 1:**
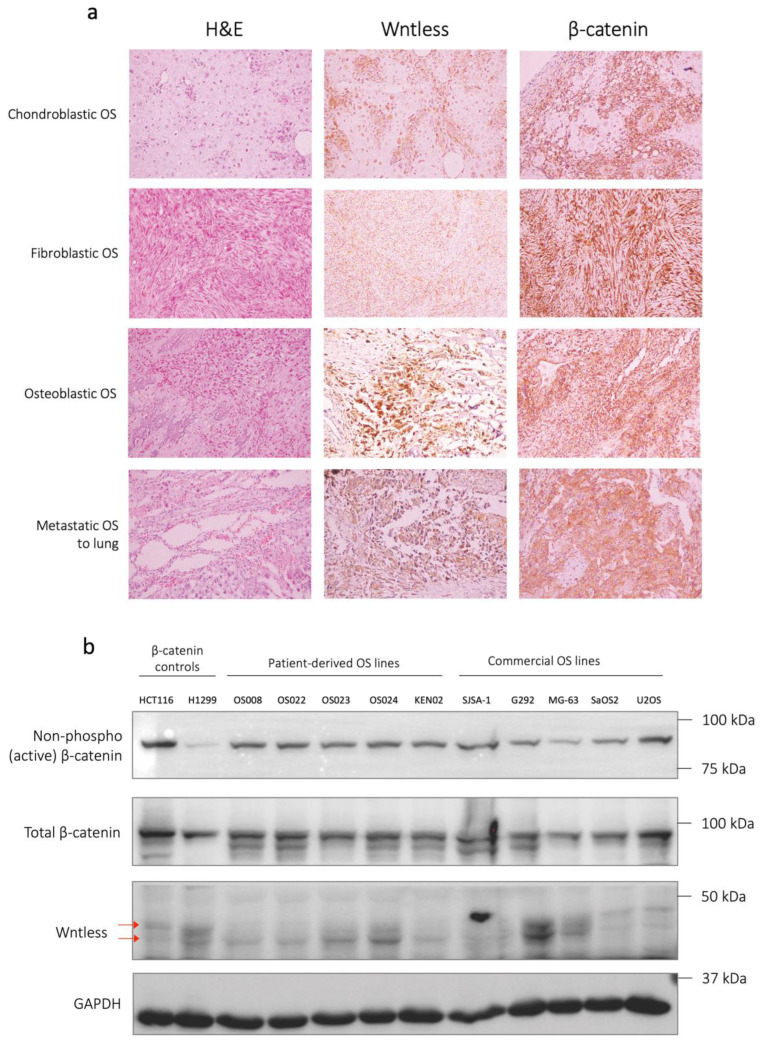
The Wnt signalling pathway is elevated in OS patient samples. (**a**) Haematoxylin and eosin (H&E) and IHC scans of clinical OS tumour samples showing strong staining of WLS and β-catenin. Images taken at 200× magnification. (**b**) Western blot performed for non-OS cell lines (HCT116 and H1299), established OS PDC lines, and commercial OS cell lines against non-phospho (active) β-catenin, total β-catenin, WLS (clone YJ5, arrows refer to the double band of WLS).

Wntless (WLS) is a multi-pass transmembrane protein which transports palmitoylated Wnts to the plasma membrane for secretion [28]. Using a novel antibody that recognizes the first extracellular loop of the human WLS protein [29], we investigated the relative abundance of WLS proteins in different subtypes of OS from patient FFPE samples. The OS samples show high WLS and β-catenin expression by IHC (Figure 1a). This indicates that the level of expression of WLS in tissue sections correlates highly with expression levels of β-catenin, suggesting that Wnt signalling is upregulated in OS.

We then established patient-derived cell (PDC) lines for analysis. These stable lines were noted to recapitulate the osteoblastic and mineralizing nature of OS (Supplementary Figure S1). Protein lysates from our established clinical cell lines were used to determine the relative abundance of active β-catenin and WLS (against housekeeping protein GAPDH), in comparison to other established and characterized OS cell lines (Figure 1b, uncropped Western blots in Supplementary Figure S2). Consistent with the high levels of β-catenin observed in FFPE samples, 18 of 18 patient-derived OS cell lines demonstrated β-catenin expression levels in Western blot comparable to that of established OS cell lines such as SJSA-1, G292, MG-63, SaOS2, and U2OS. Expression of β-catenin is also significantly higher than non-OS cell lines that have been reported to endogenously express high levels of β-catenin, such as H1299 (metastatic lung carcinoma) and HCT116 (colorectal carcinoma). Notably, cell lines that displayed karyotypic abnormalities (OS008 and OS024) show comparable levels of β-catenin, an indication that karyotypic abnormalities did not lead to an appreciable effect on levels of β-catenin expression. 

### 2.2. Effect of ETC-159, a Novel PORCN Inhibitor, on Wnt Signalling in Osteosarcoma

**Figure 2:**
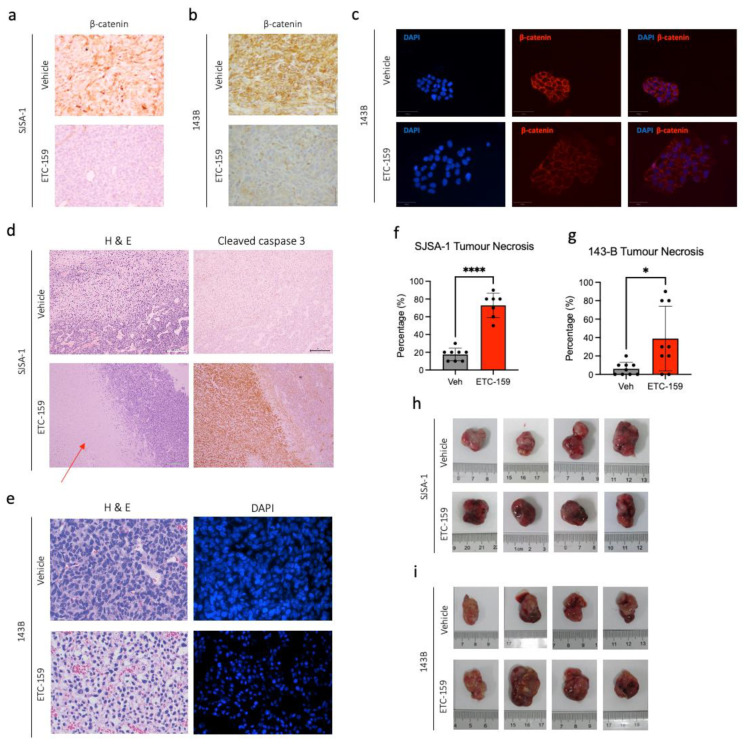
Effect of ETC-159 on osteosarcoma xenografts. Mice bearing OS xenografts were gavaged with 30 mg/kg ETC-159 daily. IHC staining in (**a**) SJSA-1 and (**b**) 143B xenograft sections show marked reduction in β-catenin following ETC-159 treatment. Images were captured at 200× magnification. (**c**) Immunofluorescence staining for β-catenin following treatment with ETC-159 shows a similar reduction. Images were captured at 100× magnification. (**d**) Decreased eosin staining in SJSA-1 xenograft sections after ETC-159 treatment. Red arrow indicates necrotic region of tumour. IHC shows increased cleaved caspase 3 (indicating apoptosis) following ETC-159 treatment. This region also overlaps with necrotic region of tumour identified by H&E staining. Images captured at 100× magnification. (**e**) H&E staining of 143B xenograft shows increased pyknotic nuclei following ETC-159 treatment and decreased eosin stain uptake due to tumour necrosis within xenograft. This is also clearly seen when nuclei are stained for DAPI. Images captured at 400× magnification. Scatter plots showing percentage tumour necrosis of tumour in (**f**) SJSA-1 or (**g**) 143B xenografts. Each point represents an individual tumour. Percentage of tumour necrosis was derived by quantifying the number of pyknotic nuclei in H&E-stained sections. * indicates *p* < 0.05, **** indicates *p* < 0.0001. Significance was calculated with unpaired two-tailed Student’s *t*-test. Increased tumour haemorrhage seen in (**h**) SJSA-1 and (**i**) 143B tumours following ETC-159 treatment.

A marked decrease in the levels of β-catenin was observed with IHC when xenografts were treated with ETC-159, indicating that ETC-159 was effective in reducing Wnt signalling both in SJSA-1 and 143B xenografts (Figure 2a,b. Photomicrographs for other treated tumour samples are available in Supplementary Figure S4). Tumour growth curves and final tumour volume are shown in Supplementary Figure S3. This was also confirmed in vitro with immunofluorescence staining (Figure 2c). Strong evidence of tumour necrosis was noted in xenograft sections, with significant increase in cleaved caspase 3 (Figure 2d), as well as prominently condensed pyknotic nuclei following ETC-159 treatment (Figure 2e). Taken together, these results suggest that treatment with ETC-159, an upstream Wnt inhibitor, can induce tumour necrosis in OS, consistent with our hypothesis that OS is a Wnt-driven neoplasm. Furthermore, we noted that ETC-159-treated xenografts show high levels of haemorrhage (Figure 2h,i), and sought to elucidate the mechanism of ETC-159 action in OS. 

### 2.3. ETC-159 Decreases Angiogenesis in Osteosarcoma 

**Figure 3:**
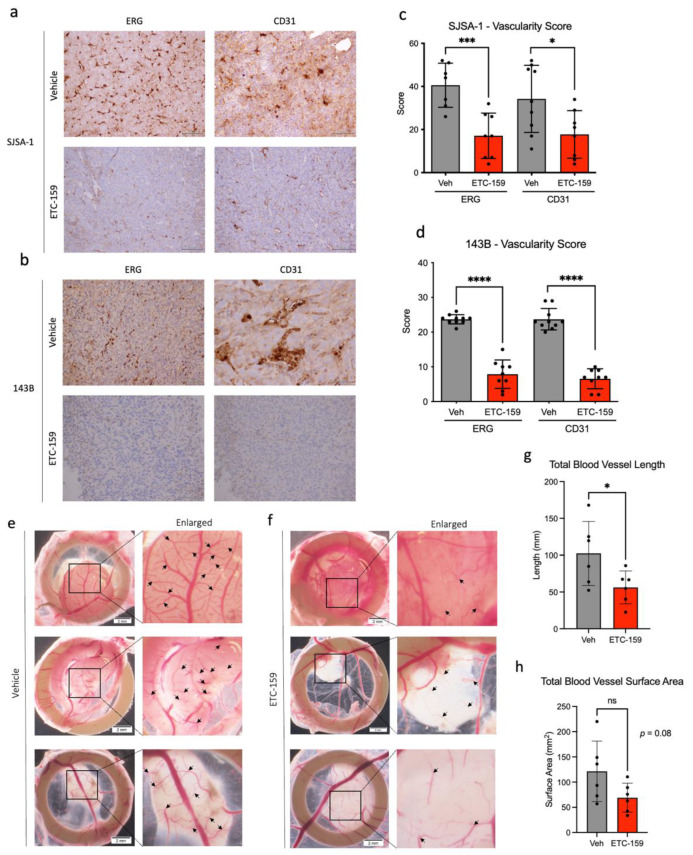
IHC of ERG and CD31 (endothelial markers) in (**a**) SJSA-1 and (**b**) 143B xenografts, showing a reduction after treatment with ETC-159. Images were captured at 200× magnification. Quantification of vascularity score in (**c**,**d**) for SJSA-1 and 143B respectively. SJSA-1 cells grafted onto CAM and treated with (**e**) vehicle or (**f**) ETC-159 (*n* = 6). Representative images of 3 independent tumours are shown, with insets magnified. Quantification of (**g**) total blood vessel length and (**h**) total blood vessel surface area. “ns” indicates not significant, * indicates *p* < 0.05, *** indicates *p* < 0.001, **** indicates *p* < 0.0001. Significance was calculated with unpaired two-tailed Student’s *t*-test.

Upon interrogating the degree of intratumor vasculature with vascular markers ERG and CD31, we noted that OS xenografts treated with ETC-159 demonstrated a striking reduction in tumour vasculature both in the degree of IHC staining (Figure 3a,c), and in the resulting vascularity score obtained from double-blinded vessel counts (Figure 3b,d). The decrease in vessel counts in the treated xenografts suggests that the mechanism of tumour necrosis previously noted following ETC-159 could potentially be due to the inhibition of angiogenic process in these OS xenograft tumours.

To validate our results in an orthogonal model, SJSA-1 cells were grafted onto chicken chorioallantoic membrane (CAM) and treated with either vehicle (Veh) or 100 nM ETC-159 (*n* = 6). These xenografts were harvested for analysis after 72 hours. Xenografts that received vehicle treatment (Figure 3e) show regular hierarchical vascular development, and treatment with ETC-159 (Figure 3f) resulted in significantly impaired vascular growth that is aberrant and dysregulated, with markedly diminished arborization of blood vessels (black arrows in Figure 3e,f, enlarged view). Total length and surface area of blood vessels around the tumour were quantified, and we note that total blood vessel length was significantly reduced following ETC-159 treatment (Figure 3g). There was also a strong trend of decrease in blood vessel surface area following ETC-159 treatment (Figure 3h), almost reaching statistical significance. This indicates that ETC-159 treatment, and therefore pan-Wnt inhibition, potently disrupts vascular formation in both CAM models, and also in murine xenografts.

### 2.4. RNAseq of Osteosarcoma Xenograft Tissue

**Figure 4:**
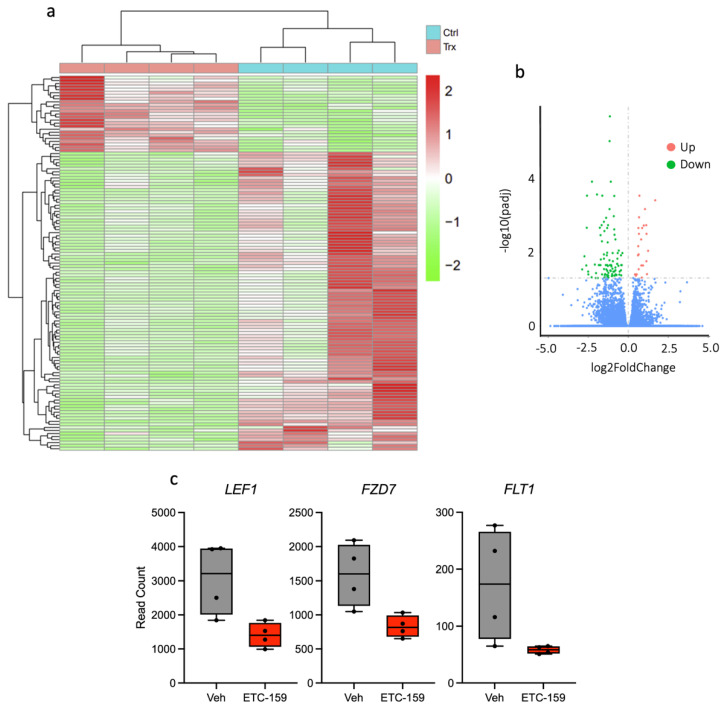
Analysis of differentially expressed genes in OS xenograft following ETC159 treatment. (**a**) A total of 123 differentially expressed genes (DEGs) with any fold change (adjusted *p*-value < 0.05) in osteosarcoma xenografts (*n* = 4) treated with ETC-159 were noted. A total of 25 genes were upregulated while 98 genes were downregulated. (**b**) Volcano plot summarizing genes differentially expressed in xenografts (*n* = 4) after treatment with ETC-159. (**c**) Significant downregulation of *LEF1*, *FZD7*, and *LEF1* in OS xenograft after treatment with ETC-159 (adj. *p*-value < 0.05). *LEF1* and *FZD7*, which are associated with Wnt signalling, were downregulated less than 2-fold, while *FLT1*, which is associated with angiogenesis, was downregulated more than 2-fold.

RNA sequencing was performed on OS xenograft to uncover the mechanism of action of ETC-159. 123 differentially expressed genes were identified (Figure 4a), and degree of upregulation or downregulation summarized in Figure 4b. In ETC-159-treated xenografts, significant downregulation of LEF1, a downstream target of β-catenin, suggests on-target effect leading to inhibition of Wnt signalling. Unexpected downregulation of FZD7, a Wnt receptor, uncovers a novel aspect of Wnt signalling inhibition by ETC-159. Significant downregulation of FLT1, also known as VEGFR1, indicates anti-angiogenic potential of the drug in OS (Figure 4c). Since ETC-159 downregulates VEGFR1 in tumours, it suggests that proliferation of intratumoral endothelial cells could have been inhibited by ETC-159. 

## 3. Discussion

In the course of identifying potential pathways to target for therapeutic purposes, a common pharmacological approach is to look upstream of dysregulated pathways for druggable targets. In the case of OS that has been shown to have an active Wnt pathway for targeting [30], examination of key components of Wnt/β-catenin signalling and their subsequent inhibition could potentially serve to uncover novel targets with untapped therapeutic value [25]. 

OS is an osteogenic tumour which produces osteoid, an essentially osteoblastic process. Elevated levels of Wnt signalling have been shown to be important in other conditions with abnormal bone formation (e.g., heterotrophic ossification, myositis ossificans, ossification of posterior longitudinal ligament). Since Wnts have been reported to be important in regulating bone formation, it would not be surprising that OS tumours possess and require intact Wnt signalling for oncogenic proliferation. Contrary to the report by Cai et al. which suggested that OS tumours are Wnt low [31], we report here that elevated levels of β-catenin are observed in the PDC lines used in this study. This is consistent with previous observations that aberrant Wnt signalling has been implicated in OS tumorigenesis [32], pointing to inhibition of the Wnt pathway as a possible therapeutic target for OS [7]. 

In this study, the authors have taken a further step to examine expression levels of WLS, a key intermediate in the Wnt signalling pathway, and have shown that WLS level is high in OS. Demonstrating that OS tumours have elevated WLS expression is important as WLS is involved in trafficking Wnt out of the cell, resulting in the eventual activation of Wnt signalling [33]. Examination of WLS expression would therefore serve as an indicator of an intact Wnt pathway, making OS amenable to ETC-159 as a potential neoadjuvant therapeutic. While other studies have identified targets downstream in the Wnt pathway for therapeutic purposes [33,34], the authors believe this is the first study using upstream WLS expression to evaluate the feasibility of Wnt inhibition in OS. As there are currently no good inhibitors of WLS, the authors chose to focus on the effects of PORCN inhibitor instead. A novel PORCN inhibitor with good bioavailability, ETC-159 inhibits Wnt signalling through the upstream inhibition of PORCN [35]. PORCN is responsible for the palmitoylation of Wnt for interaction with WLS. Without this modification, Wnt molecules cannot interact with WLS for secretion [36,37,38,39], and downstream Wnt signalling is unable to be propagated. 

For a more physiological representation of the effects of ETC-159 in OS, the authors chose to use two well-established OS cell lines, SJSA-1 and 143B, for subsequent xenograft implantation in a murine model. Work on SJSA-1 and 143B has been published and these cell lines are well characterized [25,26,27], making the results obtained in this preclinical study reproducible, and therefore preferable for use in xenograft models. These xenograft tumours demonstrated a profound on-target response to treatment with ETC-159 in vivo, with a marked decrease in tumour β-catenin levels, as evident from the IHC sections presented in Figure 2. The presence of β-catenin in control samples is an indicator of active Wnt signalling, and the reduction in tumour β-catenin demonstrates that ETC-159 is effective in inhibiting the canonical Wnt signalling pathway. In the absence of Wnt stimulus, β-catenin is rapidly targeted for proteasomal degradation [40] by the multiprotein destruction complex [41], resulting in a decrease in β-catenin levels. This can be seen in the IHC sections of ETC-159-treated tumour tissue. Interestingly, both SJSA-1 and 143B xenograft tumours demonstrated similar increase in tumour necrosis (Figure 2) and reduction in tumour vasculature (Figure 3) following ETC-159 treatment. The extent of tumour necrosis in the tumour was observable from H&E-stained sections, the increase in cleaved caspase staining in IHC, and in the visibly condensed nuclei that is typical of dying cells [42]. Cleaved caspase 3 is the most downstream effector caspase in the apoptotic process, and others have shown that it is a reliable indicator for the study of tumour necrosis. It has been reported that the presence of cleaved caspase 3 is rare in normal homeostatic cells, only increasing sharply after chemotherapeutic treatment [43]. Crowley et al. corroborate this, pointing out that activated cleaved caspase 3 is a unique signature and reliable marker to cells that are dying, undergoing apoptosis, or have already died [44]. Others have also used this marker as a readout for apoptosis in synovial sarcoma models [45]. Interestingly, despite the increase in tumour necrosis, the tumour volume did not change with treatment. OS tumours also consist of mineralized osteoid tissue and may not shrink as rapidly as other soft tissue tumours which are mostly water by volume. Tumour necrosis could also be occurring late in the treatment period, resulting in inadequate time for tumour volume shrinkage before the mice were sacrificed. The eventual effect of ETC-159 treatment is the reduction in vascularity and induction of tumour necrosis, which is of therapeutic benefit, and point toward the clinical utility of ETC-159 for the treatment of chemoresistant OS.

Wnt signalling has been shown to be a player in the regulation of angiogenesis in solid tumours [46], and mechanistic studies examining how aberrant Wnt signalling is able to directly modulate angiogenesis have been reported [18,20,21,22,23,24]. The lack of stabilized β-catenin that can enter the nucleus for transcriptional initiation following Wnt inhibition reduces overexpression of Wnt-associated pro-angiogenic genes such as Cox-2 (*PTGS2*), which stimulates endothelial cell migration [47], and *VEGF*, which has been implicated in pathological angiogenesis [48] through its ability to potently stimulate endothelial cell proliferation and differentiation. It is thus unsurprising to find that Wnt inhibition results in decreased vascularity in our treated tumour samples, and increased tumour necrosis arising from reduced blood flow. The use of an orthogonal system, the chicken chorioallantoic membrane (CAM) model, clearly recapitulates this phenotype. Due to the immunodeficient status of the developing egg, tumours can be inoculated onto the CAM surface, and changes in vascularity after drug treatment are clearly visible. RNAseq analysis of tumour tissue also confirmed the effect that ETC-159 had on OS intratumoral angiogenesis, downregulating expression of angiogenic signalling intermediate *FLT1* (Figure 4). Furthermore, increased tumour necrosis in OS tumours has been associated with prolonged survival [49]. Our observations open the way for potential use of ETC-159 for treatment of chemoresistant OS tumours.

Meanwhile, analysis of RNAseq data from publicly available dataset GSE99671 of differentially-expressed genes between osteosarcoma bone sample and normal paired samples indicated that one of the top two most significantly upregulated pathways is hypoxia (adj. *p*-val < 0.05). Since hypoxia is known to promote vessel growth by upregulating pro-angiogenic pathways [31], this result supports the involvement of active tumour vascularization in oncogenesis process of osteosarcoma. Treatment of ETC-159 in OS with higher tumour vascularization might thus allow detection of significant downregulation of angiogenesis.

It has recently been reported that ETC-159 has progressed on to clinical trials in human patients for the treatment of advanced solid tumours [50]. It is relatively well tolerated and specifically targets Wnt signalling. While long term treatment with Wnt inhibitors do result in side effects such as gastrointestinal hypoplasia and osteoporosis, preliminary trials indicate that there is a wide range of dose tolerance for ETC-159 [35], and there exist treatment strategies to address these side effects (i.e. bisphosphonate treatment for loss of bone mass [51]). This, together with promising pre-clinical data showing induction of tumour necrosis via the inhibition of angiogenesis, shows that ETC-159 may have the potential to be developed further and used in a neoadjuvant setting for tumours that are different and difficult to treat through simple mechanistic inhibition. 

## 4. Materials and Methods

### 4.1. Study Population

All samples were obtained from archival of biopsies of patients that presented with osteosarcoma in National University Hospital of Singapore (NUH) from 1 January 1995 to 15th September 2014. The NHG Domain Specific Review Board (NHG DSRB Ref: 2014/00900, NHG DSRB B/00/301) approved the study; informed consent for experimental use of the tissues was obtained.

### 4.2. Isolation and Characterization of New Osteosarcoma Cell Lines

OS cell lines (*n* = 10) were developed from OS biopsies from patients prior to chemotherapy. Stable cell lines were established as previously described [52]. Briefly, OS tissue samples were minced and propagated in DMEM. Subsequent passages were grown in mixed culture medium (comprising 9:1 (*v*/*v*) of Roswell Park Memorial Institute 1640 (RPMI-1640; Gibco, Waltham, MA, USA) medium and DMEM supplemented with 15% FBS (hereafter referred to as 15% RD medium). After the initial tumour cells reached sub-confluence levels with piled-up foci of cells, morphologically uniform colonies were selected and cell lines further passaged with no change in cell morphology or growth rate, indicating that a stable, immortal cell line had been established. 

To confirm that OS cells were osteoblastic and mineralizing, Alizarin red staining was performed. OS cells were maintained in differentiation medium (50 mg/mL ascorbic acid-2-phosphate, 10 mM β-glycerophosphate, and 100 nM dexamethasone) (Sigma, St. Louis, MS, USA) for 4 weeks, before being fixed with methanol (20 °C). Distilled water was used to rinse cultures, before staining with 0.01% Alizarin Red S (pH 5.5) for 30 min. Staining intensity was documented on an Epson Perfection photo scanner (Seiko Epson, Los Alamitos, CA, USA).

Biopsy samples from 18 distinct patients with high grade OS were also fixed in formalin and embedded in paraffin (FFPE). Sections were taken and stained with haematoxylin and eosin (H&E), and other sections stained with immunohistochemistry for relative expression of β-catenin; total β-catenin (610154, BD Biosciences, San Jose, CA, USA) and non-phosphorylated (active) β-Catenin (#8814), Cell Signaling Technology, Danvers, MA, USA) and Wntless (WLS) (MABS87, Millipore, Burlington, MA, USA).

### 4.3. Establishment of Human Tumor Xenograft in Immunodeficient Mice

Animal procedures were performed in accordance with Institutional Animal Care and Use Committee approval (AN-1407-009-230). Female 6–8 week old BALB/c nude mice were obtained from Shanghai Lingchang BioTechnology Co., Ltd., Shanghai, China. Animals were regularly monitored to ensure that they were healthy with no adverse events reported. 

A quantity of 2 × 10^6^ OS (SJSA-1 and 143B, obtained from ATCC (Manassas, VA, USA)) cells were injected subcutaneously at the right flank. Mice were inspected daily for tumour growth formation and health status. Prior to treatment, mice were randomly assigned into two groups based on initial tumour volume. Mice were either treated with vehicle (PEG-400:Water, 1:1, *n* = 10) or drug (ETC-159 in vehicle, 30 mg/kg, *n* = 10). Treatment for all mice commenced when mean tumour size reached approximately 168 mm^3^ and was administered daily for 15 days by oral gavage. Tumour volume was measured twice weekly. Tumour samples were fixed in formalin for FFPE processing, or preserved in RNA Save (Cat No. 01-891-1, Biological Industries, Beit HaEmek, Israel) for downstream applications. FFPE sections were stained with H&E and photographed using a TS2 microscope with a DS-L4-Fi3 camera system from Nikon SBIC-NIC at Biopolis, Singapore at 40× magnification. For DAPI staining, the sections were stained with UltraCruz Mounting Medium (Santa Cruz, Dallas, TX, USA) and photographed using an Olympus BX61 with a CoolSNAP HQ2 camera at 40× magnification. IHC was performed using β-catenin, cleaved caspase 3 and anti-Wntless antibodies (same as above). Double-blinded IHC scoring of all cases was performed by two different pathologists.

### 4.4. Examination of Osteogenic Markers in Patient Samples

Formalin fixed paraffin-embedded tumour tissue was sectioned (3–4 µm thick) and deparaffinized with xylene before being rehydrated in sequential ethanol gradients. The steam-pressure-cooker method was used for antigen retrieval. Sections were immersed in 10 mM citrate buffer (pH 6.0) before hydrogen peroxide was used to inactivate endogenous peroxidase activity. The sections were probed with diluted primary antibody overnight at 4 °C. An EnVision™/HRP Rabbit/Mouse detection kit (Dako, Agilent Technologies, Santa Clara, CA, USA) together with 3,3′-diaminobenzidine (DAB+) chromogen substrate was used to stain sections. Gill’s haematoxylin was used to counterstain nuclei before dehydration and mounting. Primary antibodies used include β-catenin and cleaved caspase 3 (Cell Signaling Technologies, Danvers, MA, USA). Stained sections were imaged with an Olympus BX43 microscope with a DP21 microscope camera attachment (Olympus, Tokyo, Japan). Scoring of all IHC cases was performed by two different pathologists who were double blinded to experimental conditions. Quantification of percentage tumour necrosis was done through analysing the area of necrosis with respect to total tumour surface area.

### 4.5. Immunoblot Analysis of Cell Growth Markers

Cells were lysed using 50 mM Tris-HCl, 150 mM NaCl, 1% sodium deoxycholate, 0.25 mM EDTA (pH 8.0), 1% Triton X-100, 0.2% sodium fluoride, and protease inhibitor cocktail (Sigma, Darmstadt, Germany). WLS (637304, Merck, Darmstadt, Germany), β-catenin (BD Biosciences, NJ, USA), GAPDH (Cell Signaling Technologies), anti-rabbit IgG-HRP and anti-mouse IgG-HRP secondary antibodies (Agilent Technologies, Santa Clara, CA, USA), and SuperSignal™ West Dura substrate (ThermoFisher Scientific, Waltham, MA, USA) were used to develop immunoblots on PVDF. The images were captured using the LAS-3000 Life Science Imager (Fujifilm, Tokyo, Japan).

### 4.6. Chick Chorioallantoic Membrane (CAM) Assay

All procedures were carried out in accordance with NUS IACUC guidelines and regulations. Fertilized White Leghorn chicken eggs were obtained from a local breeder, and designated as embryonic development day 0 (EDD0) upon start of incubation at 37 °C. On EDD7, inoculation of 5 × 10^6^ cells suspended in 1:1 Matrigel (50 µL total) was performed. A sterile silicone O-ring was used to contain the tumour cells after the CAM was scored using a sterile pipette tip. Tumours that formed on CAM were treated with 100 nM ETC-159 on EDD10.Tumours and a small portion of the CAM were excised and imaged with Olympus SZ51 stereomicroscope 72 h later (EDD13). Quantification of vessel length and surface area was performed using ImageJ.

### 4.7. RNA Sequencing and Data Analysis

RNA sequencing and data analysis were carried out by Novogene (https://en.novogene.com/). mRNA was purified from total RNA using poly-T magnetic beads. Following fragmentation, first-strand cDNA synthesis was carried out using random hexamer primers. Second-strand cDNA synthesis using dUTP was then performed for directional library. Qubit and bioanalyzer were used to check the library for size distribution detection. The library preparations were sequenced on an Illumina platform for the generation of paired-end reads.

Hisat2 v2.0.5 was used to align paired-end clean reads to the reference genome. featureCounts v1.5.0-p3 was used to count the reads mapped to each gene and FPKM calculated. DESeq2 (1.20.0) was used for differential expression analysis, and differentially expressed genes were analysed with Gene Ontology (GO) using the clusterProfiler R package. 

### 4.8. Data Analysis

Data were analysed using Prism v5.0 (GraphPad Software, San Diego, CA, USA). Statistical analysis was performed with the unpaired *t*-test. The significance for all tests was set at *p* ≤ 0.05.

## 5. Conclusions

OS tumours demonstrated genotype heterogeneity, which increases the difficulty in developing drugs targeting a specific mechanism. OS tumours had elevated levels of WLS and β-catenin, although there were varying levels of WLS between cell lines. This suggests that the Wnt/β-catenin pathway is characteristically upregulated in OS. Treatment with ETC-159, an upstream Wnt inhibitor, resulted in more than 40 percent increase in tumour necrosis, as well as significantly lower vascularity scores.

This study has demonstrated that ETC-159 has the potential to treat OS tumours. Interestingly, it seems to inhibit angiogenesis in these tumours. This may broaden the usage of ETC-159 across other tumour types, including those which do not undergo tumour necrosis as a direct response to Wnt inhibition. Further understanding of how ETC-159 acts on OS models will be useful in developing new and effective neoadjuvant therapeutic drugs.

## Data Availability

The datasets used and/or analysed during the current study are available from the corresponding author on reasonable request.

## Supplementary Materials

The supporting information can be downloaded at: https://www.mdpi.com/article/10.3390/ijms24054759/s1.
