# Supplementary material for: ETC-159, an Upstream Wnt inhibitor, Induces Tumour Necrosis via Modulation of Angiogenesis in Osteosarcoma
Source: Int J Mol Sci. 2023 Mar 1;24(5):4759. doi: 10.3390/ijms24054759 (PMC10003732; doi:10.3390/ijms24054759)

Supplementary Materials

- **Supplementary Figure S1.** Morphology of OS PDC lines and Alizarin Red staining. These are anonymised samples of patients diagnosed with osteosarcoma by tumour biopsy. They are a mix of both sexes and come from patients between the ages of 12 to 68. **(a)** Morphology of cell lines at 75%-90% confluence. 4x magnification. **(b)** Alizarin red assays were performed to assess mineralization of OS PDC lines. 40x magnification. **(c)** negative control for Alizarin Red staining.

- **Supplementary Figure S2.** Whole Western Blots of OS cell lines for non-phosphor (active) β-catenin, total β-catenin, Wntless, and housekeeping protein GAPDH.

- **Supplementary Figure S3.** Tumour volume growth curves and final tumour weights for SJSA-1 (a, b) and 143B xenografts (c, d).

- **Supplementary Figure S4.** IHC for β-catenin on SJSA-1 xenograft sections. Mice were treated with either **(a)** vehicle or **(b)** ETC-159. The ETC-159 treatment set showed greatly reduced β-catenin staining intensity as compared to the control group. Images were captured at 20x and 40x as indicated.

Supplementary Figure S1. PDC photomicrographs and Alizarin red staining

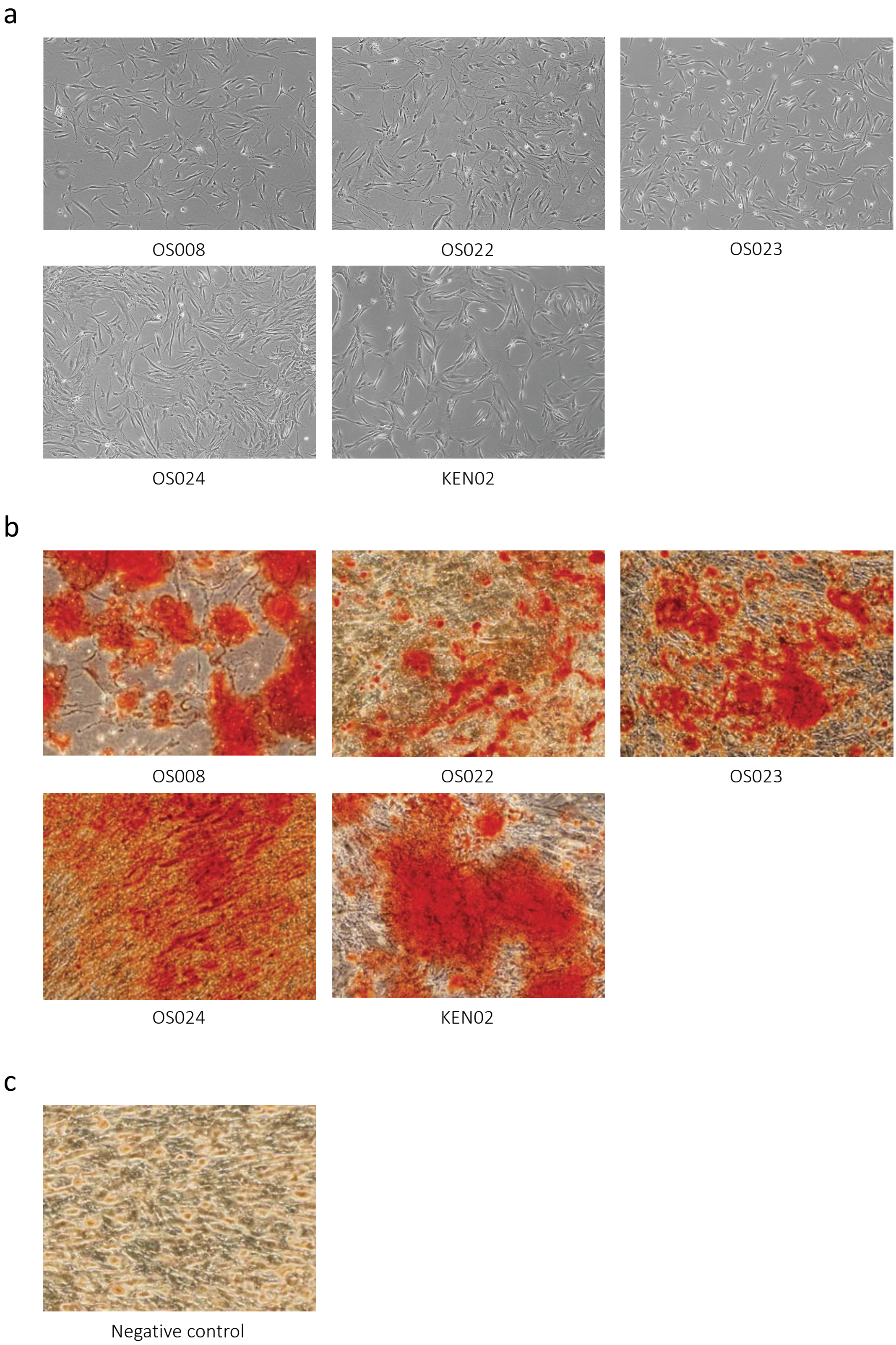

Supplementary Figure S2. Whole Western blots of OS cell lines

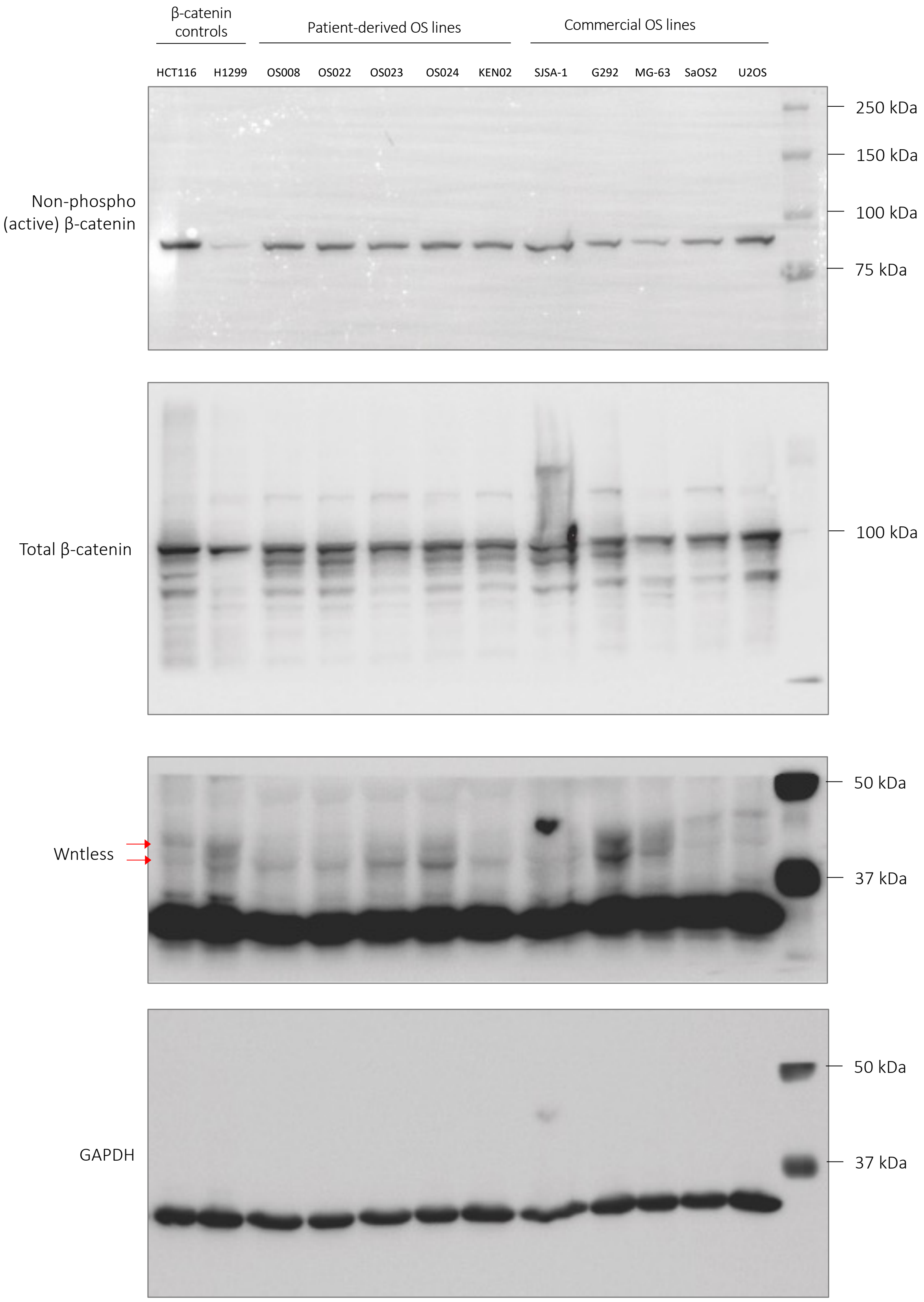

Supplementary Figure S3. Tumour growth curves and final weights

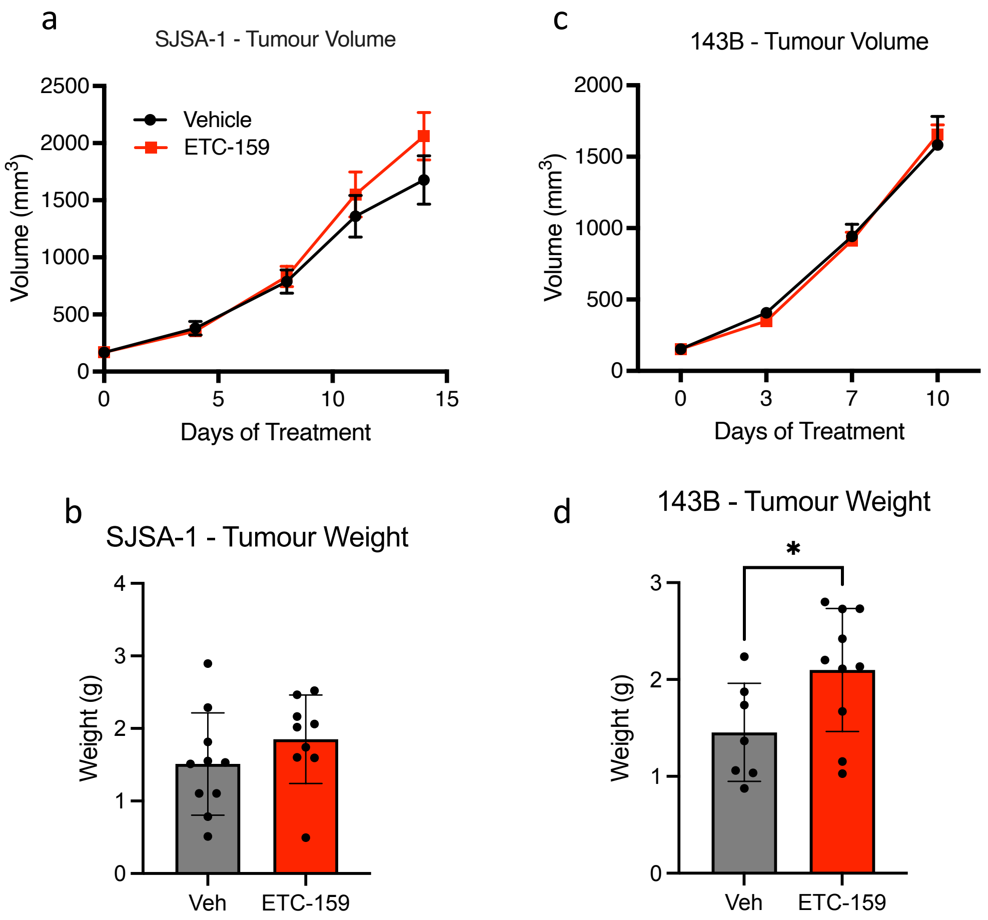

Supplementary Figure S4. IHC for β-catenin in SJSA-1 Vehicle / Treated xenografts

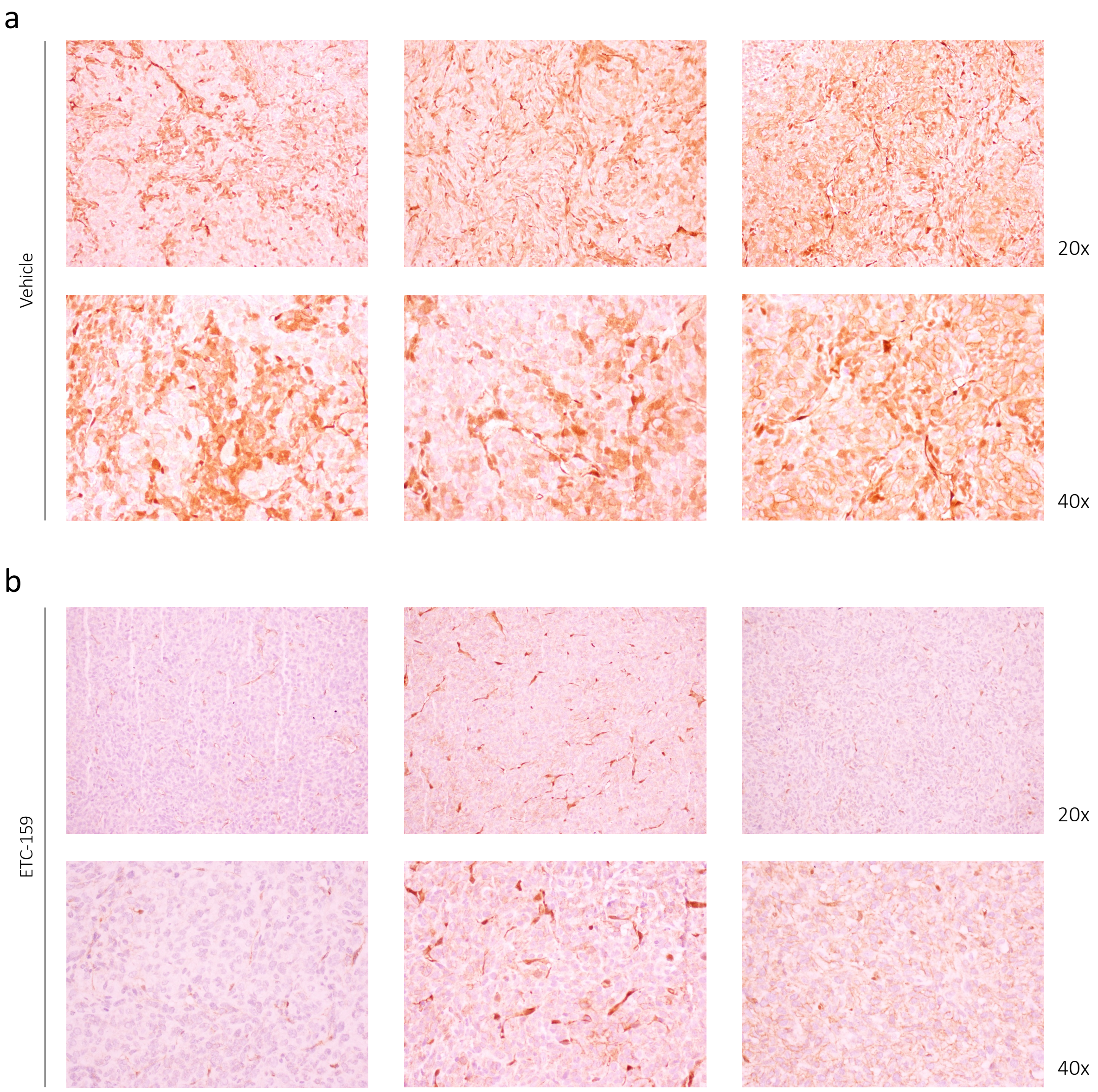

Supplement: Supplementary file 1 [file ijms-24-04759-s001.zip › ijms-2160014-supplementary.pdf]
